# Birth prevalence of neural tube defects in eastern Africa: a systematic review and meta-analysis

**DOI:** 10.1186/s12883-022-02697-z

**Published:** 2022-06-01

**Authors:** Paddy Ssentongo, Emily S. Heilbrunn, Anna E. Ssentongo, Lydia V. N. Ssenyonga, Alain Lekoubou

**Affiliations:** 1 Department of Public Health Sciences, Penn State Hershey College of Medicine and Milton S. Hershey Medical Center, Hershey, PA USA; 2 Center for Neural Engineering, Department of Engineering, Science and Mechanics, The Pennsylvania State University, State College, PA USA; 3 Department of Medicine, Penn State Hershey College of Medicine and Milton S. Hershey Medical Center, Hershey, USA; 4 Department of Surgery, Penn State Hershey College of Medicine and Milton S. Hershey Medical Center, Hershey, PA USA; 5 Department of Nursing, Faculty of Health Sciences, Busitema University, TORORO, Uganda; 6 Department of Neurology, Penn State Hershey College of Medicine and Milton S. Hershey Medical Center, Hershey, PA USA

**Keywords:** Neural tube defects, Spina bifida, Anencephaly, Encephalocele, Eastern Africa, Birth prevalence, Global health

## Abstract

**Background:**

Neural tube defects (NTDs) are associated with high rates of neonatal mortality and morbidity worldwide. The promotion of folic acid fortification and supplementation in pregnant women by the Food and Drug Administration significantly decreased the incidence of NTDs in the United States. This practice is not widely adopted in Eastern Africa countries. We hypothesized that these countries experience a higher burden of NTDs than countries that promote the use of folic acid. We aimed to estimate the birth prevalence of NTDs in the United Nations (UN) Eastern African region.

**Methods:**

PubMed (Medline), Embase, and Cochrane Library databases were systematically searched from inception to December 17, 2021. We included randomized controlled trials or observational studies that reported the prevalence estimates of NTDs in Eastern Africa. Random effects model was used to pool the effect estimates. The GRADE (Grading of Recommendations, Assessment, Development and Evaluation) approach was used to assess the certainty of the evidence. Outcome measures were overall and specific (spina bifida, anencephaly, encephalocele) rates of NTDs per 10,000 births, including live and stillborn cases.

**Results:**

The meta-analysis included 20 studies consisting of 752,936 individuals. The pooled prevalence of all NTDs per 10,000 births in Eastern Africa was 33.30 (95% CI: 21.58 to 51.34). Between-study heterogeneity was high (*I*^2^ = 97%, *p* < 0.0001), The rate was highest in Ethiopia (60 per 10,000). Birth prevalence of spina bifida (20 per 10,000) was higher than anencephaly (9 per 10,000) and encephalocele (2.33 per 10,000). No studies on NTDs were identified in 70% of the UN Eastern Africa region. Birth prevalence increased by 4% per year from 1983 to 2018. The level of evidence as qualified with GRADE was moderate.

**Conclusion:**

The birth prevalence of NTDs in the United Nations region of Eastern Africa is 5 times as high as observed in Western countries with mandatory folic acid supplementation in place. Therefore, mandatory folic acid supplementation of stable foods may decrease the risk of NTDs in Eastern Africa.

**Supplementary Information:**

The online version contains supplementary material available at 10.1186/s12883-022-02697-z.

## Background

Neural tube defects (NTDs) are the second most common birth defects in humans arising as a result of incomplete closure of the brain or spinal cord in the 3rd and/or 4th week of pregnancy [1]. Nearly 200,000 neonates are estimated to be born each year with NTDs in low- and middle-income countries (LMICs) [2]. Despite decades-old evidence that dietary supplementation with folic acid (FA) significantly decreases cases of NTDs and spina bifida, in particular, [3] many sub-Saharan African countries have yet to mandate folic acid fortification of their grain products [4]. Among children born with NTDs, mortality is over 75% before 5 years [5]. In addition to causing stillbirths, neonatal, infant, and under-five mortality, NTDs significantly contribute to disability-adjusted life years and increases health care costs among survivors [6].

After randomized clinical trials demonstrated the efficacy of periconceptional supplements with FA in preventing NTDs, [3, 7] the U.S. Public Health Service recommended that all women capable of becoming pregnant consume 400 μg of folic acid daily to prevent neural tube defects [8]. In 1996, the United States Food and Drug Administration required that manufacturers add 140 *μ*g folic acid per 100 g of grain product labeled as enriched. As a result, NTDs prevalence decreased from 10.7 per 10,000 live births in 1995–1996 (before fortification) to 7.0 per 10,000 in 2009–2011 (after fortification) [9]. However, in Eastern Africa, such mandates of fortification of grain do not exist or are not enforced [10]. Furthermore, in Eastern Africa, most women do not realize that they are pregnant until after 28 days of gestation when the neural tube has closed. A critical timing to start FA supplementation is missed as pregnant women start taking the supplements in the second trimester when they attend antenatal care. In addition, women from lower socioeconomic status cannot afford relatively expensive foods such as dairy and meat products, which are rich sources of folic acid [11]. Eastern Africa has one of the highest prevalences of human immunodeficiency virus/acquired immunodeficiency syndrome (HIV/AIDS) in the world [12]. Current recommendations for the treatment of HIV during pregnancy include taking antiretroviral therapy (ART) [13]. However, ART has been associated with spinal tube defects recently [14–16]. Taken together, these factors warrant determining the current prevalence rates of NTD in Eastern Africa to inform public health programs and policy and research activities.

To date, systematic reviews estimating the prevalence of NTDs have focused on high-income countries, in part due to a lack of studies in LMICs such as sub-Saharan Africa [5]. Even with such increased risk factors of NTDs, we are not aware of population-based surveillance programs for NTDs in Eastern Africa. However, hospital-based surveillance programs are steadily increasing with a subsequent increase in the number of publications on the prevalence of NTDs. Therefore, we conducted a systematic review and meta-analysis to 1) estimate the birth prevalence of NTDs in Eastern Africa, 2) describe country-level differences in prevalence, and 3) establish a temporal trend for the birth prevalence.

## Methods

### Search strategy and selection criteria

This study is being reported per the reporting guidance provided in the 2020 Preferred Reporting Items for Systematic Reviews and Meta-Analyses (PRISMA) statement and Meta-analysis of Observational Studies in Epidemiology (MOOSE) [17, 18]. See completed PRISMA checklist in **Supplementary Table** **1**. We searched PubMed (Medline), Embase, and Cochrane Library databases from inception to December 17, 2021. We also searched the grey or difficult-to-locate literature. We performed hand-searching of the reference lists of included studies, relevant reviews, or other relevant documents using Google Scholar. No limitations were imposed based on study design and language. The search terms of interest included Medical Search Headings (MeSH) and keywords—“congenital abnormalities” OR “neural tube defects” OR “anencephaly” OR” encephalocele” OR “spina bifida” OR “meningocele” OR “myelomeningocele” AND [“Uganda” OR “Kenya” OR “Tanzania” OR “Ethiopia” OR “Malawi” OR “Eritrea” OR “Burundi” OR “Comoros” OR “Djibouti” OR “Madagascar” OR “Mauritius” OR “Mayotte” OR “Mozambique” OR “Reunion” OR “Rwanda” OR “Seychelles” OR “Somalia” OR “South Sudan” OR “Zambia” OR “Zimbabwe”]. For a full list of search terms, see **Supplementary Table** **2**. Duplicate studies were initially extracted via Endnote software. Two reviewers (ESH and AES) independently screened titles and abstracts of the studies for inclusion eligibility. The comprehensive list of studies from our initial search was transferred into Endnote, which further removed duplicate studies. The inclusion criteria for this meta-analysis and systematic review was defined as 1) observational studies (cohort, cross-sectional, nested case-control) and randomized controlled trials reporting the incidence or prevalence estimates with and without confidence bounds of neural tube defects (encephalocele, spina bifida, or anencephaly), 2) conducted in human subjects, 3) conducted in Eastern African countries listed above, 4) population-based (all cases in a defined geographical area, or hospital or community-based surveillance). We excluded studies 1) not conducted in human subjects, 2) did not report the rates of NTDs or congenital anomalies, 3) meta-analyses, 4) literature reviews, and 5) commentaries.

### Data extraction and quality assessment

After the reviewers initially screened titles and abstracts of potential articles, full-text articles were independently screened by two reviewers (ESH and AE) for eligibility. If duplicate articles were identified, we included only mutually exclusive data. All included articles were scored for methodological quality using the quality assessment tool for NTDs published by Atta et al. (2016) [4]. The GRADE (Grading of Recommendations, Assessment, Development and Evaluation) approach was used to assess the certainty (quality) of the evidence. Discrepancies between the reviewers were resolved by discussion until consensus was reached. However, if an agreement was not reached, a third independent reviewer (PS) was involved in the discussion until consensus was reached. Articles that met inclusion criteria had appropriate data extracted using a standard data collection form. We extracted the following information: the year of publication, country, year of study, study design, sample size, proportion male, the proportion with HIV, number of births with anencephaly, spina bifida, and encephalocele. Incidence and prevalence estimates were all classified as prevalence because, as discussed by Mason et al., prevalence is the preferred measure of the frequency of birth defects epidemiology [19]. Due to high rates of pregnancy loss induced by congenital defects, the observed rates suffer from selection bias and do not fully represent the true incidence.

### Statistical analysis

All statistical analyses were performed with R software version 3.6.2 (R Project for Statistical Computing). The primary outcome was the birth prevalence of NTDs reported per 10,000 total informative births (live births only or live births + stillbirths only or live births + stillbirths + spontaneous abortions). The *metaprop* function from the R package *meta* was used to calculate the pooled effect estimates [20]. Meta-analysis was performed with the DerSimonian-Laird random-effects model with Hartung-Knapp-Sidik-Jonkman variance correction [21]. Individual and pooled estimates were graphically displayed using forest plots. A random-effects model assumes the observed estimates of NTDs can vary across studies because of real differences in the effect in each study and sampling variability (chance). Between-study heterogeneity was assessed using *I*^2^ statistics, expressed as % (low (25%), moderate (50%), and high (75%) and Cochrane’s *Q* statistic (significance level < 0.05) [22]. To investigate the sources of heterogeneity, we conducted a subgroup analysis estimating NTDs by country in which the study was conducted. Potential ascertainment bias (as might be caused by publication bias) was assessed with funnel plots by plotting the study effect size against standard errors of the effect size and Egger’s test [23].

## Results

### Study selection

**Fig. 1:**
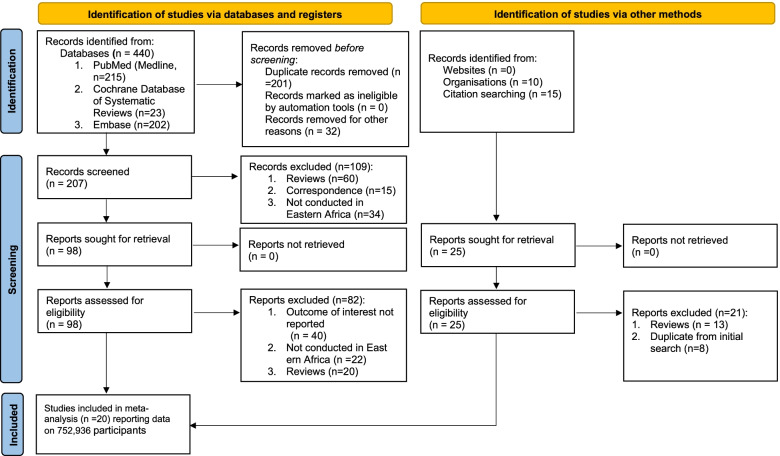
PRISMA flow diagram for study selection

Our initial searches yielded 465 studies, of which 123 underwent full-text screening (Fig. 1). Of these, 20 studies were eligible for inclusion. Of the included studies, nine were conducted in Ethiopia, [24–32] three from Tanzania, [33–35] three from Uganda, [36–38] three from Kenya, [39–41] and one from Malawi and Eritrea each [42, 43]. The total sample size of this meta-analysis and systematic review was 752,936 individuals. No studies were identified from the rest of the United Nations region of Eastern Africa—Burundi, Comoros, Djibouti, Madagascar, Mauritius, Mayotte, Mozambique, Reunion, Rwanda, Seychelles, Somalia, South Sudan, Zambia, and Zimbabwe. Wu et al. (2013) and Mosha et al. (2014) were community-based surveillance studies, but the rest were hospital-based surveillance. The median study quality score for studies reporting on the incidence or prevalence of NTDs was 5 out of 6 (range = 4–6). Study-specific details and references are given in Table 1.

**Table 1:** Study-specific details and references

Study	Country	Surveillance	NTD	Sample Size	Rate Per 10,000	Anencephaly	Spina Bifida	Encephalocele	Denominator	Quality Score
Mumpe-Mwanja et al., 2019 [36]	Uganda	Hospital-Based	72	69,766	10.3				live births, stillbirths and sponteneous abortion	6
Ndibazza et al., 2011 [37]	Uganda	Hospital-based	2	2365	8.5	1	1	0	live births and stillbirths	5
Ochieng 2011 [38]	Uganda	Hospital-based	3	754	39.8	0	3	0	live births	4
Onyambu et al., 2018 [39]	Kenya	Hospital-based	3	500	60.0	1	0	2	live births and stillbirths	5
Wu et al., 2013 [40]	Kenya	Community-based	5	5559	8.99	0	3	2	living children	5
Mosha et al., 2014 [33]	Tanzania	Community-based	1	1783	5.6	0	1	0	live births, stillbirths and sponteneous abortion	6
Muga et al., 2009 [41]	Kenya	Hospital-based	15	7355	20.4	10	4	1	live births and stillbirths	5
Kishimba et al., 2015 [34]	Tanzania	Hospital-based	27	27,230	9.9	14	10	3	live births	6
Gedefaw et al., 2018 [28]	Ethiopia	Hospital-based	55	8677	63.4	15	35	3	live births and stillbirths	5
Berihu et al., 2018 [29]	Ethiopia	Hospital-based	195	14,903	130.9	99	96		live births and stillbirths	6
Taye et al., 2019 [27]	Ethiopia	Hospital-based	304	76,201	39.9	36	268		living children (0–17 y)	6
Geneti et al., 2019 [31]	Ethiopia	Hospital-based	186	45,951	40.5	63	33	4	live births and stillbirths	6
Seyoum & Adane, 2018 [30]	Ethiopia	Hospital-based	103	19,650	52.4	7	53		live births and stillbirths	6
Sorri & Mesfin, 2015 [24]	Ethiopia	Hospital-based	117	28,961	40.4	77	95	5	live births and stillbirths	4
Taye et al., 2016 [26]	Ethiopia	Hospital-based	1873	319,776	58.6	163	995	25	living children (0–17 y)	4
Msamati et al., 2000 [43]	Malawi	Hospital-based	12	25,562	4.7		12		live births	5
Kinasha and Manji, 2002 [35]	Tanzania	Hospital-based	103	34,000	30.3	4	89	10	live births	5
Estifanos et al., 2017 [42]	Eritrea	Hospital-based	185	39,803	46.5	75	27		live births and stillbirths	6
Mekonen et al., 2015 [25]	Ethiopia	Hospital-based	20	1516	131.9	1	19		live births	5
Abdu & Seyoum, 2019 [32]	Ethiopia	Hospital-based	121	22,624	53.5		121		live births	5

### Pooled birth prevalence of neural tube defect events per 10,000 observations

**Fig. 2:**
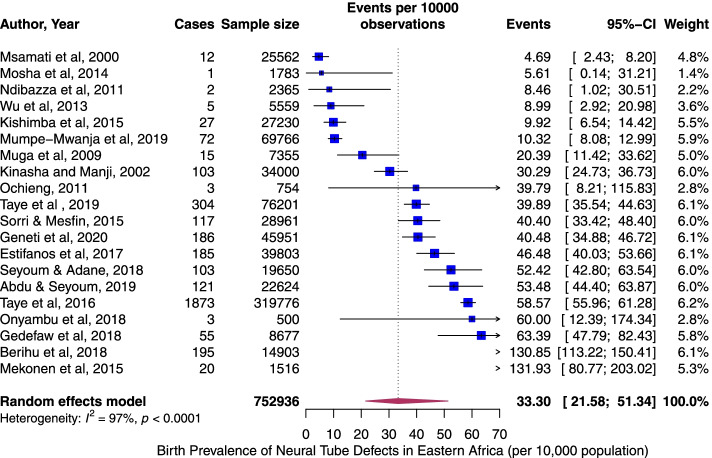
Overall pooled birth prevalence of all neural tube defects per 10,000 births

**Fig. 3:**
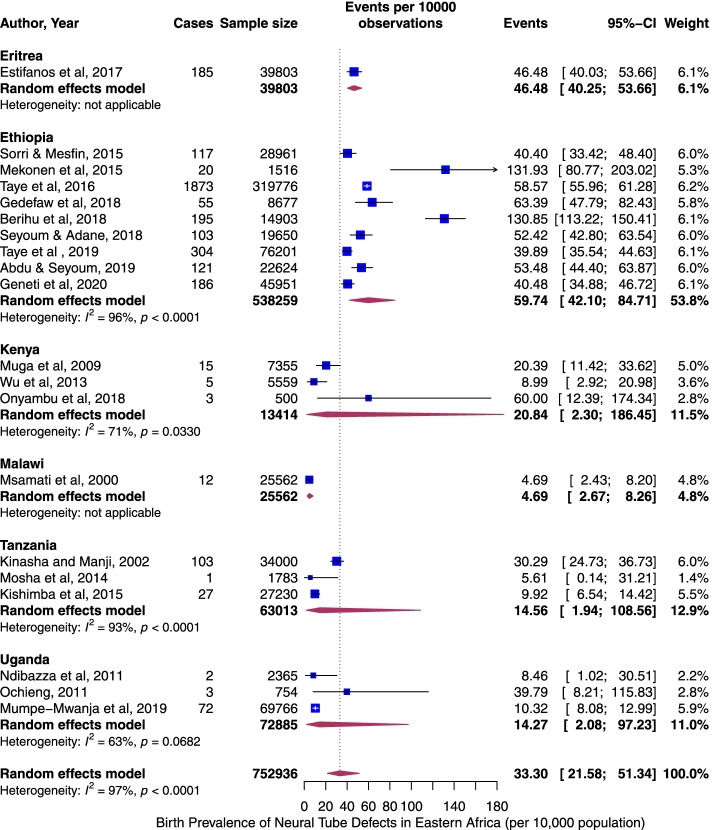
The pooled birth prevalence of neural tube defects stratified by country

**Fig. 4:**
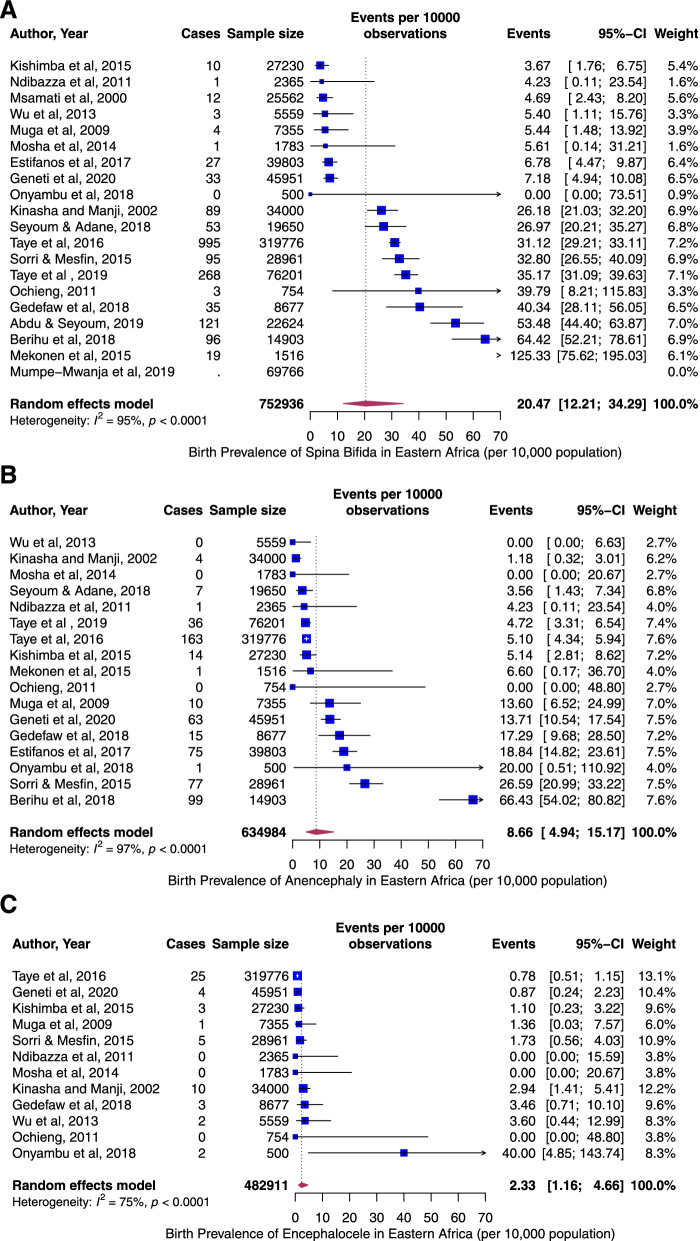
**A** The pooled birth prevalence of spina bifida per 10,000 births. **B**: The pooled birth prevalence of anencephaly per 10,000 births. **C**: The pooled birth prevalence of encephalocele per 10,000 births

**Fig. 5:**
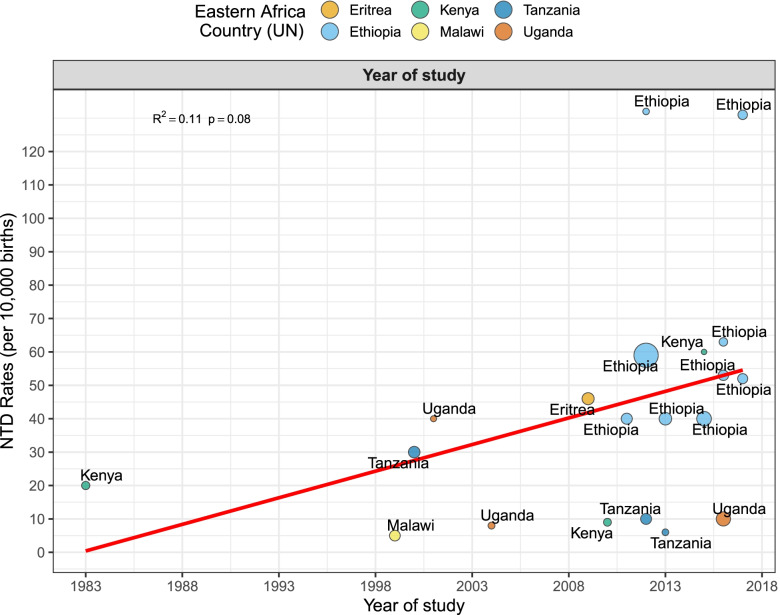
Temporal trend in the incidence of NTDs in Eastern Africa. The birth prevalence of NTDs increased at a rate of 4% per year from 1983 to 2018. Linear fit from linear regression model. Circles and color represent countries. The size of the circle is proportional to the sample size of each study

The pooled period prevalence of all neural tube defect events per 10,000 observations was 33.30 (95% CI: 21.58 to 51.34). Between-study heterogeneity was high (*I*^2^ = 97%, *p* < 0.0001), Fig. 2). To assess country-specific differences in the pooled rates of NTDs, a sub-group meta-analysis stratified by country was conducted (Fig. 3). Ethiopia demonstrated the highest birth prevalence of NTDs, 59.74 (95% CI: 42.10 to 84.71, *I*^2^ = 96%, *p* < 0.0001), followed by Eriteria. The rate was lowest in Malawi, 4.69 (95% CI: 2.67 to 8.26, *I*^2^ = NA). Next, we assess specific NTDs—Spina bifida, anencephaly, and encephalocele. Among the NTDs, spina bifida rate was the highest, 20.47 per 10,000 (95% CI: 12.21 to 34.29, *I*^2^ = 95%, *p <* 0.0001, Fig. 4A) followed by anencephaly; 8.66 (95% CI: 4.94 to 15.17, *I*^2^ = 97%, *p <* 0.0001, Fig. 4B) and encephaloceles were reported less frequently, 2.33 per 10,000 (95% CI: 1.16 to 4.66, *I*^2^ = 75%, *p <* 0.0001, Fig. 4C). Using the median year of study (not publication year), and starting from 1983, the rate of NTDs increased by 4% per year (*p* = 0.07) (Fig. 5).

### Sensitivity analysis and publication Bias

To assess the potential for outlier and influential studies affecting the robustness of the pooled estimates, we conducted influence sensitivity analyses for the birth prevalence of NTDs (**Supplementary Fig.** **1****)**, spina bifida (**Supplementary Fig.** **2****)**, **encephalocele (Supplementary Fig.** **3****) and anencephaly (Supplementary Fig.** **4****) separately** [44]. In this meta-analysis, a named study was omitted and replaced one study at a time (leave-one-out method) from the meta-analysis and recalculated the pooled estimates for the remaining studies. The estimates remained close to the overall pooled estimate, indicating that no individual study significantly influenced the pooled estimate. The funnel plot and the value of the Egger’s test (*p* = 0.04) revealed the presence of publication bias in the reporting of the overall NTDs (**Supplementary Fig.** **5****)**. Trim and fill analysis was conducted to adjust for the potential publication bias. Analyses suggest that the adjusted effect estimates would fall in the range of 33 to 92 per 10,000 births, and 7 additional studies were added to the funnel plots (**Supplementary Fig.** **6****).**

## Discussion

In this meta-analysis, the pooled birth prevalence of neural tube defects was 33 (95% CI: 22 to 51). The pooled prevalence of NTDs (60 per 10,000 births) was highest in Ethiopia and lowest in Malawi (5 per 10,000 births). The estimated point prevalence of spina bifida was two times higher than anencephaly and ten times higher than encephalocele.

The pooled prevalence of NTDs in Eastern Africa is nearly five times as high as that of the post-stratification era of the United States. In the United States, the transition to fortifying foods with folic acid substantially decreased the prevalence of NTDs in newborns. The Centers for Disease Control and Prevention reports that the proportion of babies with NTDs has decreased by 35% due to this implementation [9]. While this change in legislation has ultimately benefited the United States population, this practice has not been adopted in all regions across the globe. The fortification of grains and other food products in Eastern Africa has not been widely adopted and may contribute to the growing birth prevalence of NTDs in the region.

Recently, a study of birth defects in infants born to women with HIV infection in Botswana reported an eightfold increased risk for NTDs among births with periconceptional exposure to ART that included integrase inhibitor dolutegravir (DTG) compared with other ART regimens [14]. Although a recent surveillance study conducted in the United States did not find a difference in the birth prevalence of NTDs in HIV-exposed live births from that of the general population, [45] it is possible that other confounders such as socioeconomic status may differ between Eastern Africa and the United States study population. Considering the high prevalence of HIV in Eastern Africa and the use of ART during the periconceptional period, it is plausible that these factors could contribute to the high birth prevalence of NTDs in this region. Furthermore, the high poverty rates, lack of NTDs awareness from public health campaigns could be the driving factors of the high prevalence of NTDs in Eastern Africa.

The results of this meta-analysis contribute to renewing the call for urgent actions to curb down NTDs burden in Eastern Africa. Effective public health interventions can be conceptualized at the patient/provider or a much larger regional or national level. Increasing awareness among patients and medical providers, including those in charge of prenatal care on the issue of NTDs, may translate into more use of folic acid periconceptually. This approach seems effortful and less likely to reach a sizable population at risk. Ideally, it should be preceded by recommendations from health authorities on the daily requirement of folic acid to achieve adequate NTDs prevention. Recommended daily intake for the primary prevention of NTDs among low-risk women before conception and throughout lactation is 400 micrograms internationally [46]. A more pragmatic approach would require fortifying commonly used dietary products in each country. The experience of universal iodization has proven that such an epidemiological approach was feasible and efficient with most countries worldwide achieving iodization of at least 95% of all salt supplies. As a result, iodine deficiency, the most important preventable cause of acquired intellectual disability, has been virtually eliminated [47]. The successful elimination of iodine deficiency required the commitment of world political leaders. Similarly, fortifying foods with folic acid is more likely to be successful with the implication of local-, regional- and country-level political and healthcare stakeholders.

In the context of priority-based budgeting, financing nutrient fortification programs with folic acid require demonstrating the strategy’s cost-effectiveness. Although such data are scarce in Eastern African countries, estimates from the United States where the program has been successfully implemented are compelling. For example, after folic acid fortification, between 600 and 700 babies were born each year without spina bifida, translating into $400 million to $600 million saved every year in the United States [48]. Altogether, identifying universally consumed nutrients in Eastern Africa or each specific country and fortifying them with folic acid appears to offer the best chances to reduce the burden of NTDs.

### Public health implications of our findings

Establishing national and regional registries and harmonizing and improving diagnostic criteria would result in a better estimation of the disease burden. Some populations at risk will need special attention, including childbearing age women with epilepsy. They will need to be systematically identified and followed up, preferably by providers with epilepsy-care experience through a well-structured referral system. The prevalence of the use of antiepileptic drugs with high teratogenic potential will need to be established. Additionally, besides benefiting from fortified nutrients, those women will need to take folic supplements peri-conceptually. Finally, for children born with NTDs, prompt referral to specialized centers will need to be made as soon as the disease is detected.

#### Strengths and weakness

The results of the present meta-analysis should be interpreted in the context of potential limitations. First, the current meta-analysis relied on the quality of published literature. Cases of neural tube defects may be difficult to ascertain due to neuro-imaging equipment and neurologist/neurosurgeon scarcity. Therefore, the estimates of the current meta-analysis are likely conservative; the true incidence of NTDs in Eastern Africa is probably much higher. Second, various Eastern African countries were combined to provide pooled estimates of NTDs. Potential differences in genetics and diet across these geographic regions could have introduced variations in the estimates. Nevertheless, random-effects models were adopted to control for the possible difference in effect estimates. Lastly, only 6 countries out of 20 countries in the United Nations Eastern African region were represented in the current meta-analysis. Generalizing the current estimates to the rest of Eastern Africa countries should be done with caution. The strength of our meta-analysis lies in the literature search rigor in more than two databases and the robust statistical methods we applied. Therefore, these estimates are informed by the best, most up-to-date, and most diversified data available and can be used to guide future assessment of the economic burden of NTDs in Eastern Africa.

## Conclusion

The prevalence of NTDs in Eastern Africa countries is strikingly high compared to other regions across the globe. One may hypothesize that this can be attributed to a lack of widely accepted practice involving fortifying foods with folic acid or promoting folic acid supplements for pregnant individuals. Interventions related to folic acid supplementation or fortification need to be implemented in these regions urgently to reduce the burden of NTDs in neonates.

## Supplementary Information

**Additional file 1.**

## Data Availability

The R code and datasets analyzed during the current study are available in the GitHub (https://github.com/ssentongojeddy/Neural-Tube-Defects-in-Eastern-Africa).

## Abbreviations

NTDs: Neural tube defects
CI: Confidence Interval
GRADE: Grading of Recommendations, Assessment, Development and Evaluation
UN: United Nations
LMICs: Low- and middle-income countries
FA: Folic acid
HIV/AIDS: Human immunodeficiency virus/Acquired immunodeficiency syndrome
ART: antiretroviral therapy
PRISMA: Preferred Reporting Items for Systematic Reviews and Meta-Analyses
MOOSE: Meta-analysis of Observational Studies in Epidemiology
DTG: Integrase inhibitor dolutegravir
