# Supplementary material for: Birth prevalence of neural tube defects in eastern Africa: a systematic review and meta-analysis
Source: BMC Neurol. 2022 Jun 1;22:202. doi: 10.1186/s12883-022-02697-z (PMC9158202; doi:10.1186/s12883-022-02697-z)
Supplement: Supplementary file 1 — Additional file 1. [file 12883_2022_2697_MOESM1_ESM.docx]

**Supplementary documents to**

**Birth Prevalence of Neural Tube Defects in Eastern Africa: A Systematic Review and Meta-Analysis**

**Paddy Ssentongo, MD, PhD, MPH^1,2^§, Emily S. Heilbrunn, BS^1^ Anna E. Ssentongo, DrPH, MPH^1,3^, Lydia VN Ssenyonga, MPH^4^, Alain Lekoubou, MD, Msc^1, 5^**

^1^Department of Public Health Sciences, Penn State Hershey College of Medicine and Milton S. Hershey Medical Center, Hershey, Pennsylvania, United States of America

^2^Center for Neural Engineering, Department of Engineering, Science and Mechanics, The Pennsylvania State University, Pennsylvania, United States of America

^3^Department of Surgery, Penn State Hershey College of Medicine and Milton S. Hershey Medical Center, Hershey, Pennsylvania, United States of America

^4^Department of Nursing, Faculty of Health Sciences, Busitema University, Uganda

^5^Department of Neurology, Penn State Hershey College of Medicine and Milton S. Hershey Medical Center, Hershey, Pennsylvania, United States of America

**§To whom correspondence should be addressed:**

Paddy Ssentongo, MD, MPH, PhD

Assistant Professor

Department of PubliC Health Sciences

90 Hope Drive

Penn State College of Medicine

Hershey PA, 17033 USA

| **Section and Topic** | **Item #** | **Checklist item** | **Location where item is reported** |
| --- | --- | --- | --- |
| **TITLE** | | |  |
| Title | 1 | Identify the report as a systematic review. | Page 1 |
| **ABSTRACT** | | |  |
| Abstract | 2 | See the PRISMA 2020 for Abstracts checklist. | Page 2&3 |
| **INTRODUCTION** | | |  |
| Rationale | 3 | Describe the rationale for the review in the context of existing knowledge. | Page 4 |
| Objectives | 4 | Provide an explicit statement of the objective(s) or question(s) the review addresses. | Page 5 |
| **METHODS** | | |  |
| Eligibility criteria | 5 | Specify the inclusion and exclusion criteria for the review and how studies were grouped for the syntheses. | Page 6 |
| Information sources | 6 | Specify all databases, registers, websites, organisations, reference lists and other sources searched or consulted to identify studies. Specify the date when each source was last searched or consulted. | Page 6 |
| Search strategy | 7 | Present the full search strategies for all databases, registers and websites, including any filters and limits used. | Page 6 |
| Selection process | 8 | Specify the methods used to decide whether a study met the inclusion criteria of the review, including how many reviewers screened each record and each report retrieved, whether they worked independently, and if applicable, details of automation tools used in the process. | Page 7 |
| Data collection process | 9 | Specify the methods used to collect data from reports, including how many reviewers collected data from each report, whether they worked independently, any processes for obtaining or confirming data from study investigators, and if applicable, details of automation tools used in the process. | Page 7 |
| Data items | 10a | List and define all outcomes for which data were sought. Specify whether all results that were compatible with each outcome domain in each study were sought (e.g. for all measures, time points, analyses), and if not, the methods used to decide which results to collect. | Page 7 |
|  | 10b | List and define all other variables for which data were sought (e.g. participant and intervention characteristics, funding sources). Describe any assumptions made about any missing or unclear information. | Page 7 |
| Study risk of bias assessment | 11 | Specify the methods used to assess risk of bias in the included studies, including details of the tool(s) used, how many reviewers assessed each study and whether they worked independently, and if applicable, details of automation tools used in the process. | Page 8 |
| Effect measures | 12 | Specify for each outcome the effect measure(s) (e.g. risk ratio, mean difference) used in the synthesis or presentation of results. | Page 8 |
| Synthesis methods | 13a | Describe the processes used to decide which studies were eligible for each synthesis (e.g. tabulating the study intervention characteristics and comparing against the planned groups for each synthesis (item #5)). | Page 8 |
|  | 13b | Describe any methods required to prepare the data for presentation or synthesis, such as handling of missing summary statistics, or data conversions. | Page 8 |
|  | 13c | Describe any methods used to tabulate or visually display results of individual studies and syntheses. | Page 8 |
|  | 13d | Describe any methods used to synthesize results and provide a rationale for the choice(s). If meta-analysis was performed, describe the model(s), method(s) to identify the presence and extent of statistical heterogeneity, and software package(s) used. | Page 8 |
|  | 13e | Describe any methods used to explore possible causes of heterogeneity among study results (e.g. subgroup analysis, meta-regression). | Page 8 |
|  | 13f | Describe any sensitivity analyses conducted to assess robustness of the synthesized results. | Page 8 |
| Reporting bias assessment | 14 | Describe any methods used to assess risk of bias due to missing results in a synthesis (arising from reporting biases). | Page 8 |
| Certainty assessment | 15 | Describe any methods used to assess certainty (or confidence) in the body of evidence for an outcome. | Page 8 |
| **RESULTS** | | |  |
| Study selection | 16a | Describe the results of the search and selection process, from the number of records identified in the search to the number of studies included in the review, ideally using a flow diagram. | Page 9 |
|  | 16b | Cite studies that might appear to meet the inclusion criteria, but which were excluded, and explain why they were excluded. | Fig 1 |
| Study characteristics | 17 | Cite each included study and present its characteristics. | Table 1 |
| Risk of bias in studies | 18 | Present assessments of risk of bias for each included study. | Table 1 |
| Results of individual studies | 19 | For all outcomes, present, for each study: (a) summary statistics for each group (where appropriate) and (b) an effect estimate and its precision (e.g. confidence/credible interval), ideally using structured tables or plots. | Fig 2,3,4 |
| Results of syntheses | 20a | For each synthesis, briefly summarise the characteristics and risk of bias among contributing studies. | Page 9&10 |
|  | 20b | Present results of all statistical syntheses conducted. If meta-analysis was done, present for each the summary estimate and its precision (e.g. confidence/credible interval) and measures of statistical heterogeneity. If comparing groups, describe the direction of the effect. | Page 9&10 |
|  | 20c | Present results of all investigations of possible causes of heterogeneity among study results. | Supp |
|  | 20d | Present results of all sensitivity analyses conducted to assess the robustness of the synthesized results. | Supp |
| Reporting biases | 21 | Present assessments of risk of bias due to missing results (arising from reporting biases) for each synthesis assessed. | Supp |
| Certainty of evidence | 22 | Present assessments of certainty (or confidence) in the body of evidence for each outcome assessed. | Fig2,3,4 |
| **DISCUSSION** | | |  |
| Discussion | 23a | Provide a general interpretation of the results in the context of other evidence. | Page 11 |
|  | 23b | Discuss any limitations of the evidence included in the review. | Page 12 |
|  | 23c | Discuss any limitations of the review processes used. | Page 12 |
|  | 23d | Discuss implications of the results for practice, policy, and future research. | Page 12 |
| **OTHER INFORMATION** | | |  |
| Registration and protocol | 24a | Provide registration information for the review, including register name and registration number, or state that the review was not registered. | Not registered |
|  | 24b | Indicate where the review protocol can be accessed, or state that a protocol was not prepared. | Not reported |
|  | 24c | Describe and explain any amendments to information provided at registration or in the protocol. | NA |
| Support | 25 | Describe sources of financial or non-financial support for the review, and the role of the funders or sponsors in the review. | Page 15 |
| Competing interests | 26 | Declare any competing interests of review authors. | Page 15 |
| Availability of data, code and other materials | 27 | Report which of the following are publicly available and where they can be found: template data collection forms; data extracted from included studies; data used for all analyses; analytic code; any other materials used in the review. | Page 15 |

**Supplemental Table 1: 2020 PRISMA Checklist**

| **Database** | **Search Terms** |
| --- | --- |
| **Medline** | 1. “ congenital abnormalities”).mp. [mp=title, abstract, original title, name of substance word, subject heading word, keyword heading word, protocol supplementary  concept, rare disease supplementary concept, unique identifier]  2. ( neural tube defects ).mp. [mp=title,  abstract, original title, name of substance word, subject heading word, keyword heading word,  protocol supplementary concept, rare disease supplementary concept, unique identifier]  3. (anencephaly).mp. [mp=title,  abstract, original title, name of substance word, subject heading word, keyword heading word,  protocol supplementary concept, rare disease supplementary concept, unique identifier]  4. (encephalocele).mp. [mp=title,  abstract, original title, name of substance word, subject heading word, keyword heading word,  protocol supplementary concept, rare disease supplementary concept, unique identifier]  5. (spina bifida).mp. [mp=title,  abstract, original title, name of substance word, subject heading word, keyword heading word,  protocol supplementary concept, rare disease supplementary concept, unique identifier]  6. (meningocele).mp. [mp=title,  abstract, original title, name of substance word, subject heading word, keyword heading word,  protocol supplementary concept, rare disease supplementary concept, unique identifier]  7. (myelomeningocele).mp. [mp=title,  abstract, original title, name of substance word, subject heading word, keyword heading word,  protocol supplementary concept, rare disease supplementary concept, unique identifier]  8. (“Uganda” OR “Kenya” OR “Tanzania” OR “Ethiopia” OR “Malawi” OR “Eritrea” OR “Burundi” OR “Comoros” OR “Djibouti” OR “Madagascar” OR “Mauritius” OR “Mayotte” OR “Mozambique” OR “Reunion” OR “Rwanda” OR “Seychelles” OR “Somalia” OR “South Sudan” OR “Zambia” OR and “Zimbabwe”).mp. [mp=title, abstract, original title, name of substance word, subject heading word, keyword heading word, protocol supplementary concept, rare disease supplementary concept, unique identifier]  9. 1 or 2 or 3 or 4 or 5 or 6 or 7  10. 9 and 8 |
| **EMBASE** | 1. “ congenital abnormalities”).mp. [mp=title, abstract, original title, name of substance word, subject heading word, keyword heading word, protocol supplementary  concept, rare disease supplementary concept, unique identifier]  2. ( neural tube defects ).mp. [mp=title,  abstract, original title, name of substance word, subject heading word, keyword heading word,  protocol supplementary concept, rare disease supplementary concept, unique identifier]  3. (anencephaly).mp. [mp=title,  abstract, original title, name of substance word, subject heading word, keyword heading word,  protocol supplementary concept, rare disease supplementary concept, unique identifier]  4. (encephalocele).mp. [mp=title,  abstract, original title, name of substance word, subject heading word, keyword heading word,  protocol supplementary concept, rare disease supplementary concept, unique identifier]  5. (spina bifida).mp. [mp=title,  abstract, original title, name of substance word, subject heading word, keyword heading word,  protocol supplementary concept, rare disease supplementary concept, unique identifier]  6. (meningocele).mp. [mp=title,  abstract, original title, name of substance word, subject heading word, keyword heading word,  protocol supplementary concept, rare disease supplementary concept, unique identifier]  7. (myelomeningocele).mp. [mp=title,  abstract, original title, name of substance word, subject heading word, keyword heading word,  protocol supplementary concept, rare disease supplementary concept, unique identifier]  8. (“Uganda” OR “Kenya” OR “Tanzania” OR “Ethiopia” OR “Malawi” OR “Eritrea” OR “Burundi” OR “Comoros” OR “Djibouti” OR “Madagascar” OR “Mauritius” OR “Mayotte” OR “Mozambique” OR “Reunion” OR “Rwanda” OR “Seychelles” OR “Somalia” OR “South Sudan” OR “Zambia” OR and “Zimbabwe”).mp. [mp=title, abstract, original title, name of substance word, subject heading word, keyword heading word, protocol supplementary concept, rare disease supplementary concept, unique identifier]  9. 1 or 2 or 3 or 4 or 5 or 6 or 7  10. 9 and 8 |
| **Cochrane Library** | 1. “ congenital abnormalities”).mp. [mp=title, abstract, original title, name of substance word, subject heading word, keyword heading word, protocol supplementary  concept, rare disease supplementary concept, unique identifier]  2. ( neural tube defects ).mp. [mp=title,  abstract, original title, name of substance word, subject heading word, keyword heading word,  protocol supplementary concept, rare disease supplementary concept, unique identifier]  3. (anencephaly).mp. [mp=title,  abstract, original title, name of substance word, subject heading word, keyword heading word,  protocol supplementary concept, rare disease supplementary concept, unique identifier]  4. (encephalocele).mp. [mp=title,  abstract, original title, name of substance word, subject heading word, keyword heading word,  protocol supplementary concept, rare disease supplementary concept, unique identifier]  5. (spina bifida).mp. [mp=title,  abstract, original title, name of substance word, subject heading word, keyword heading word,  protocol supplementary concept, rare disease supplementary concept, unique identifier]  6. (meningocele).mp. [mp=title,  abstract, original title, name of substance word, subject heading word, keyword heading word,  protocol supplementary concept, rare disease supplementary concept, unique identifier]  7. (myelomeningocele).mp. [mp=title,  abstract, original title, name of substance word, subject heading word, keyword heading word,  protocol supplementary concept, rare disease supplementary concept, unique identifier]  8. (“Uganda” OR “Kenya” OR “Tanzania” OR “Ethiopia” OR “Malawi” OR “Eritrea” OR “Burundi” OR “Comoros” OR “Djibouti” OR “Madagascar” OR “Mauritius” OR “Mayotte” OR “Mozambique” OR “Reunion” OR “Rwanda” OR “Seychelles” OR “Somalia” OR “South Sudan” OR “Zambia” OR and “Zimbabwe”).mp. [mp=title, abstract, original title, name of substance word, subject heading word, keyword heading word, protocol supplementary concept, rare disease supplementary concept, unique identifier]  9. 1 or 2 or 3 or 4 or 5 or 6 or 7  10. 9 and 8 |

**Supplemental Table 2: Literature search strategy**


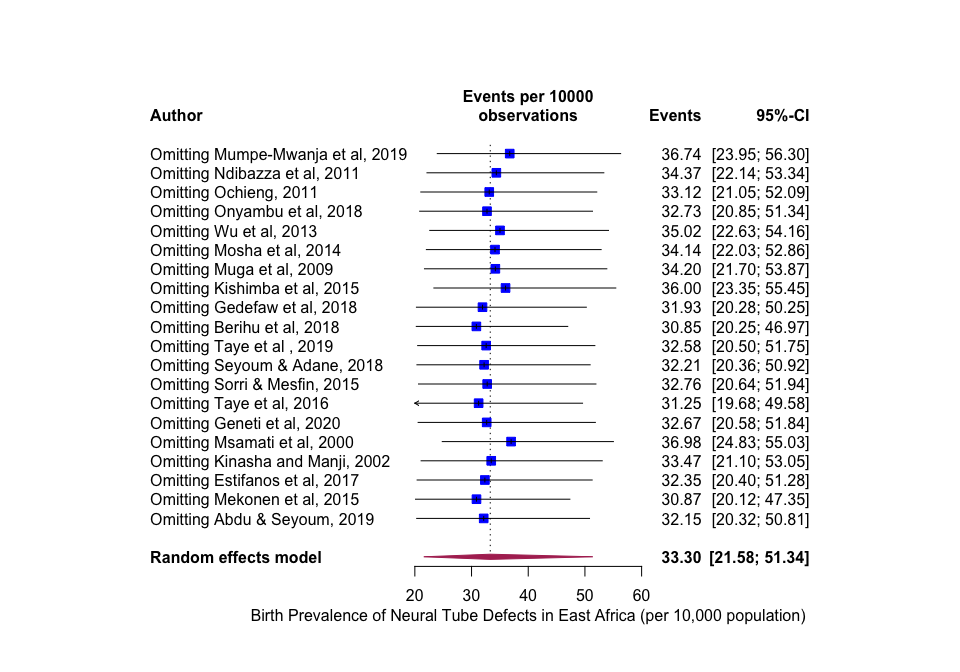


**Supplementary Figure 1: Influence and outlier (leave-one-out meta-analysis) analysis for the birth prevalence of neural tube defects (all combined).^1^ The** results of our outlier and influence analysis show the recalculated pooled point estimate ranged from 36.98 to 30.85 when one study omitted each time.


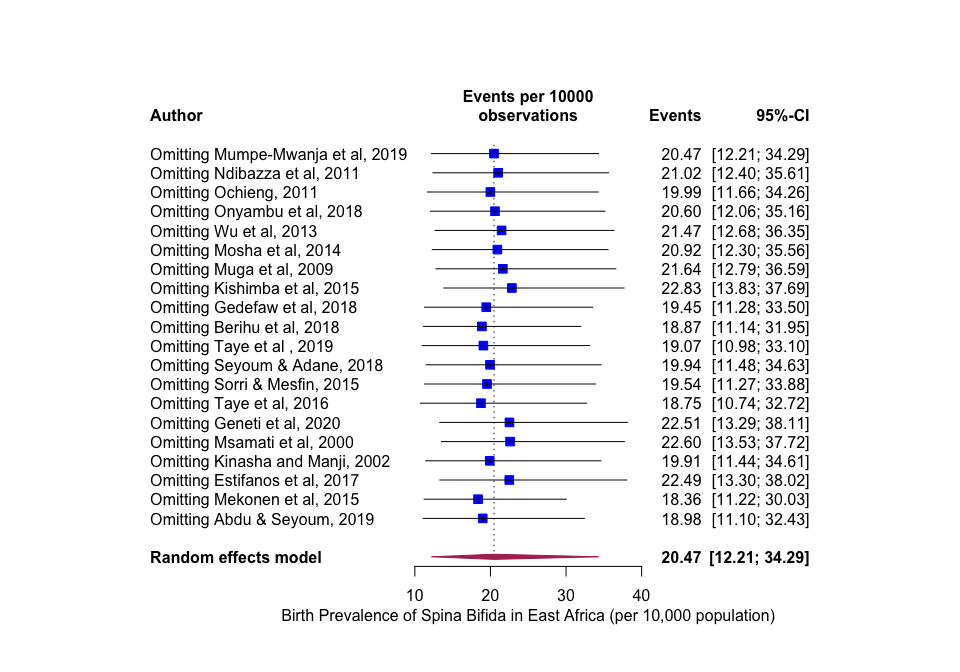


**Supplementary Figure 2: Influence and outlier (leave-one-out meta-analysis) analysis for the birth prevalence of spina bifida.^1^ The** results of our outlier and influence analysis show the recalculated pooled point estimate ranged from 22.83 to 18.36 per 10,000 population when one study omitted each time.


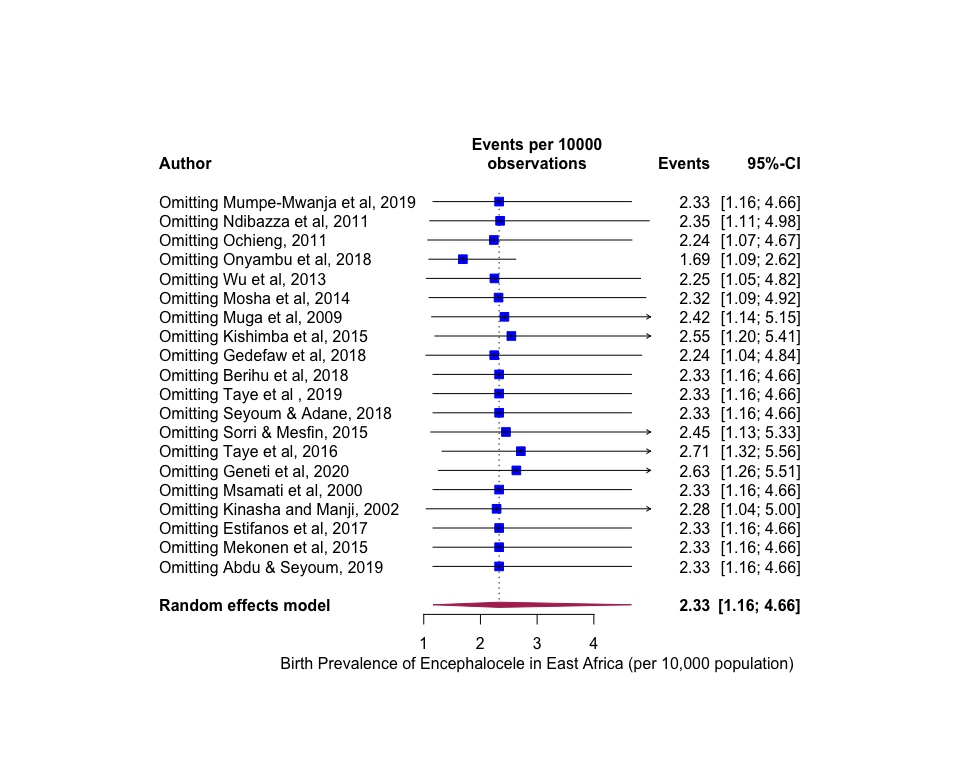


**Supplementary Figure 3: Influence and outlier (leave-one-out meta-analysis) analysis for the birth prevalence of encepahlocele.^1^ The** results of our outlier and influence analysis show the recalculated pooled point estimate ranged from 1.69 to 2.71 per 10,000 births when one study omitted each time.


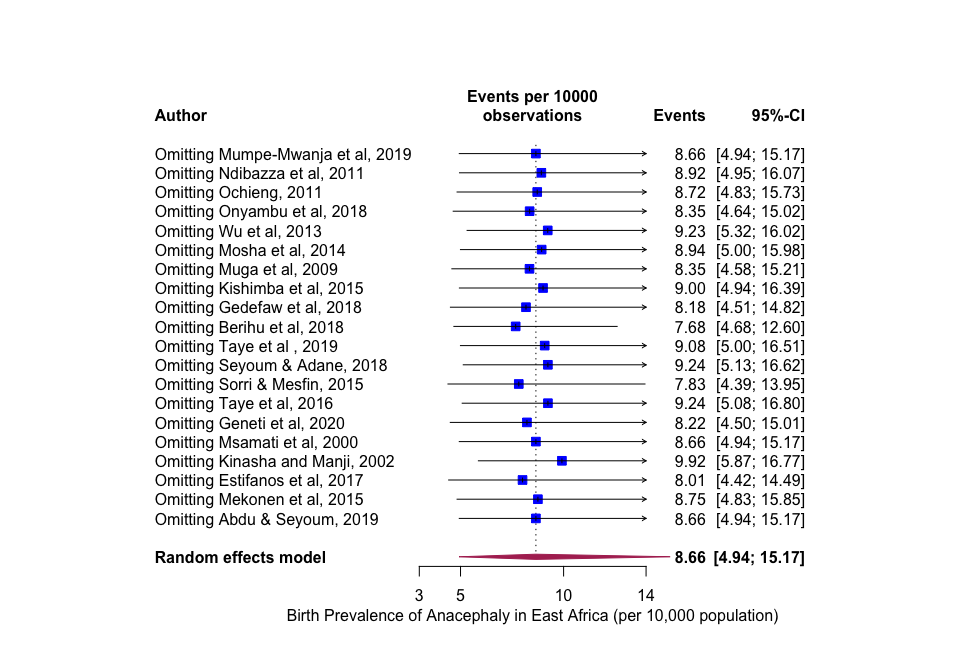


**Supplementary Figure 4: Influence and outlier (leave-one-out meta-analysis) analysis for the birth prevalence of anacephaly.^1^ The** results of our outlier and influence analysis show the recalculated pooled point estimate ranged from 7.83 to 9.24 per 10,000 births when one study omitted each time.

**Supplementary Figure 5:** **Funnel plots to assess potential for small-study publication bias.^2^ A**symmetrical inverted funnel plot suggested presence of publication bias.

**Supplementary Figure 6:** **Funnel plots from trim and fill analysis**. Duval & Tweedie trim and fill analytical method suggests that the adjusted effect estimates would fall in the range of 33 to 92 per 10,000 births and 7 additional studies were added (open circles).^3^

1. Patsopoulos NA, Evangelou E, Ioannidis JP. Sensitivity of between-study heterogeneity in meta-analysis: proposed metrics and empirical evaluation. *International journal of epidemiology* 2008; **37**(5): 1148-57.

2. Sterne JA, Becker BJ, Egger M. The funnel plot. *Publication bias in meta-analysis: Prevention, assessment and adjustments* 2005: 75-98.

3. Duval S, Tweedie R. Trim and fill: a simple funnel‐plot–based method of testing and adjusting for publication bias in meta‐analysis. *Biometrics* 2000; **56**(2): 455-63.
